# The effect of a hydrolyzed protein diet on the fecal microbiota in cats with chronic enteropathy

**DOI:** 10.1038/s41598-022-06576-y

**Published:** 2022-02-17

**Authors:** Aarti Kathrani, Sandi Yen, Jonathan R. Swann, Edward J. Hall

**Affiliations:** 1 Royal Veterinary College, Hawkshead Lane, Hertfordshire, AL9 7TA UK; 2 Oxford Centre for Microbiome Studies, Kennedy Institute of Rheumatology, University of Oxford, Oxford, OX3 7FY UK; 3 School of Human Development and Health, Faculty of Medicine, University of Southampton, Southampton, SO16 6YD UK; 4 Department of Surgery and Cancer, Sir Alexander Fleming Building, South Kensington Campus, London, SW7 2AZ UK; 5 Bristol Veterinary School, University of Bristol, Langford, Bristol, BS40 5DU UK

**Keywords:** Bacteria, Dysbiosis

## Abstract

The effect of a hydrolyzed protein diet on the fecal microbiota has not been studied in feline chronic enteropathy (CE). Our study aimed to (1) compare the fecal microbiota of cats with CE to control cats with no gastrointestinal signs and (2) determine the effect of a hydrolyzed protein diet on the fecal microbiota of cats with CE and whether this differs between dietary responders and non-responders. The fecal microbiome of cats with CE (n = 36) showed decreased α-diversity in terms of genus richness (*P* = 0.04) and increased β-diversity in terms of Bray–Curtis Dissimilarity (*P* < 0.001) compared to control cats (n = 14). *Clostridium* was the only genera significantly over-represented in cats with CE compared to control cats (adjusted *P* < 0.1). After 6-weeks of feeding the diet, fifteen cats were classified as responders and 18 as non-responders, based on clinical signs. At the genus level, α-diversity was increased in non-responders versus responders at diagnosis, but decreased after dietary intervention in both groups (*P* < 0.05). At the family level, non-responders became increasingly dissimilar after dietary intervention (*P* = 0.012). In general, the abundance of bacteria decreased with feeding a hydrolyzed diet, with the genera most significantly affected being more frequently observed in non-responders. *Bifidobacterium* was the only genus that increased significantly in abundance post-diet and this effect was observed in both responders and non-responders. Both *Oscillibacter* and *Desulfovibrionaceae_unclassified* were most abundant in non-responders at diagnosis but were rarely observed post diet in neither responders nor non-responders. Cats with CE had similar microbiota changes to those described in human inflammatory bowel disease. Whether the presence of *Oscillibacter* and *Desulfovibrionaceae_unclassified* are indicators of non-response to the diet at diagnosis requires further investigation. Despite the hydrolyzed diet reducing α-diversity in all cats with CE, this did not resolve gastrointestinal signs in some cats. However, responders metabolized the diet in a similar manner, reflected by sustained β-diversity, while the microbiome of non-responders became increasingly dissimilar compared to diagnosis at the family level. Therefore, the microbiome may not be as tightly regulated in cats with CE that are non-responders and therefore, these cats would require additional therapy for remission of clinical signs.

## Introduction

Feline chronic enteropathy (CE) describes a spectrum of diseases resulting in idiopathic chronic gastrointestinal (GI) signs^1^. Although, the pathophysiology is not well established, it is hypothesized to involve the interplay of four key components; genetic susceptibility, environmental risk factors, altered GI mucosal immune response and aberrant gut microbial composition^2^.

Gut microbial disruptions have been documented in humans with inflammatory bowel disease (IBD) and dogs with CE^3–8^. However, it is currently unknown if these changes are the cause or consequence of the chronic inflammation. Although, studies in cats have characterized the fecal microbiome, most have been in cats with acute or chronic diarrhea, where the underlying disease process was not confirmed^9,10^. However, one recent study in cats with confirmed CE documented the presence of fecal dysbiosis, with changes similar to those found in humans with IBD^11^. Therefore, further studies analyzing the fecal microbiota of cats with CE are needed to allow for a more comprehensive understanding of the GI environment and the potential benefit of dietary therapies aimed at modulating this intestinal population.

Currently, the definitive diagnosis of CE in cats requires intestinal histopathology and treatment often requires a sequential or combination approach with diet, antibiotics and immunosuppressive drugs depending on the severity of the disease^2^. Cats with chronic gastrointestinal signs without gastrointestinal biopsies performed, but where diagnostic investigations do not reveal a cause are commonly referred to as suspected CE and treated empirically. Cats with confirmed or suspected CE that respond to dietary therapy alone are described as having a food-responsive enteropathy (FRE) and are thought to comprise the majority of CE cases. Commercial hydrolyzed protein, limited ingredient novel protein, highly digestible or high fiber diets have all been used successfully for feline CE^12–19^. Although, only one small published study specifically evaluated the response to hydrolyzed protein diets in cats with CE^19^, this category of diets is frequently used for CE in cats^20^. Remission induced by a hydrolyzed protein diet in dogs with CE is accompanied by alterations in microbial community structure marked by a decreased abundance of pathobionts (*e.g.*, *Escherichia coli* and *Clostridium perfringens*) and reduced severity of dysbiosis^21^. Similar studies are unavailable for cats with CE. As such, determining the microbial and metabolic effects of a hydrolyzed protein diet in cats with CE may provide insight into diet-induced remission of GI disease in this species, which could then help to guide the development of more effective therapeutic diets.

Unfortunately, cats with FRE cannot be differentiated from CE requiring steroid treatment (SRE) based on history, clinical signs, laboratory parameters or the severity of GI endoscopic or intestinal histopathologic lesions^22^. Therefore, a therapeutic food trial is currently needed to differentiate the two conditions. The ability to differentiate FRE from SRE in cats at the time of diagnosis or shortly after may help to avoid unnecessary exclusion diet trials and delay in effective treatment to which the cat may respond. In pediatric patients with Crohn’s disease, the composition of the fecal microbiota was found to differ between responders and non-responders one week after exclusive enteral nutrition^23^. This highlights the promise of the microbiota as early predictors of response to dietary therapy. Therefore, further studies are needed to determine if the fecal microbiota at diagnosis or during dietary therapy may help to predict responders versus non-responders to a therapeutic hydrolyzed protein diet trial in cats with CE. This study sought to compare the fecal microbiota of cats with CE to the fecal microbiota of control cats with no GI signs and to determine the effect of a hydrolyzed protein diet on the fecal microbiota of cats with CE. The potential of the pre therapeutic diet fecal microbiota to discriminate between dietary responders and non-responders was also explored.

## Materials and methods

### Recruitment of case and control cats

Cats referred to the Langford Vets Small Animal Hospital, University of Bristol and Royal Veterinary College (RVC), University of London for persistent or intermittent GI signs (vomiting and/or diarrhea) of at least 2 weeks duration and where CE was suspected based on thorough investigation were considered for the study. Those cats where a commercial therapeutic hydrolyzed protein diet (Royal Canin Veterinary Diet Feline Hypoallergenic dry food (composition: rice, hydrolysed soya protein isolate, animal fats, vegetable fibers, minerals, hydrolysed poultry liver, soya oil, beet pulp, fish oil, fructo-oligosaccharides, borage oil, marigold extract (source of lutein). Key values per 100 g as fed: protein 25.5 g, fat 20 g, carbohydrate 34.5 g, dietary fiber 8.2 g, metabolizable energy 410 kcal)) trial was then used for treatment without concurrent antimicrobial and immunosuppressive therapy were prospectively recruited. All cats underwent the same diagnostic investigations consisting of a minimum of complete blood count, serum biochemistry with electrolytes, serum cobalamin (with or without folate) concentration and trans-abdominal ultrasound examination. Feline pancreatic lipase immunoreactivity (PLI, *n* = 15), trypsin-like immunoreactivity (TLI, *n* = 24), serum thyroxine (total T4, *n* = 18), basal cortisol (*n* = 0), pre- or pre and post-prandial bile acid (*n* = 16) concentrations, fecal parasitology using saturated zinc sulfate flotation (*n* = 17), fecal culture (for *Salmonella, Campylobacter* and *Clostridium difficile*, *n* = 9), empirical deworming (*n* = 9), polymerase chain reaction for *Tritrichomonas foetus* (*n* = 15) and feline leukemia and feline immunodeficiency virus testing (*n* = 13) were performed in some cats as indicated by the history, physical examination and ultrasound examination findings. Cats that had histopathologic confirmation of CE via intestinal biopsy specimens collected via endoscopy or exploratory laparotomy were included as confirmed CE, whereas those that had no intestinal biopsies performed, but investigations described above revealed no underlying cause for the chronic GI signs were included as suspected CE. The feline chronic enteropathy activity index (FCEAI) was calculated for all cats with suspected or confirmed CE^24^.

Adult cats (at least 1 year of age) without a history of GI signs (*n* = 14) and owned by staff members were recruited as controls.

The University of Bristol and Royal Veterinary College granted ethical approval for the study (VIN/14/017 and URN 2018 1837-3, respectively). Written informed consent for participation into the study was obtained from all owners of cats.

### Dietary recommendations for cats with CE

All cats with suspected or confirmed CE were discharged from the hospital with the same therapeutic hydrolyzed protein diet (Royal Canin Veterinary Diet Feline Hypoallergenic dry food). All owners were instructed to feed the therapeutic diet exclusively for 6 weeks and were specifically instructed against feeding treats and other foods. However, owners were not specifically instructed against the use of oral flavoured medication, such as deworming medication or toothpaste. Owners were asked to follow the feeding instructions on the pet food label according to current body weight and to adjust daily calories to maintain current body weight and condition if the cat was at an ideal condition or over-conditioned and to achieve and then maintain an ideal body condition if the body condition score was 3/9 or below^25^. Control cats were not given any dietary intervention.

### Fecal collection

For each cat with suspected or confirmed CE, naturally voided feces were collected during hospitalization and frozen immediately. The owners of all cats with suspected or confirmed CE were asked to schedule a recheck appointment after receiving the hydrolyzed protein diet for 6 weeks or were asked to collect feces at home at that time point if they were unable to return to the referral hospital. For those cats that returned, the owner brought naturally voided feces from the cat to the appointment, which they had frozen immediately and then transported, on ice packs to the referral hospital. For those cats that were unable to return, but owners were able to collect feces at home, samples were stored in multiple plastic bags in the freezer at the owners’ homes and transported to their local veterinary practice on ice for collection by one of the authors (A.K.). All samples were transported back to the referral hospital on dry ice.

For the control cats, all owners collected feces at home. They were frozen immediately and brought to the referral practice using ice packs.

### Assessing response to hydrolyzed protein diet in cats with CE

Owners of cats with suspected or confirmed CE were contacted via email, telephone or in person at their scheduled 6 week recheck visit to assess the response of the cat to the hydrolyzed protein diet after 6 weeks of feeding. Responders were those cats where the owners reported a significant reduction in GI signs, which did not warrant any change or additional therapy. Non-responders included those cats that were reported to have no or partial response necessitating discontinuation of the hydrolyzed protein diet with a change to a different therapeutic diet or continuation of the hydrolyzed protein diet with the addition of prednisolone, or further diagnostic investigations, such as GI biopsy if not previously performed.

### 16S rRNA gene amplicon sequencing of fecal microbiota

Fecal samples were thawed and extracted using the Qiagen PowerFecal Pro DNA kit (Qiagen #51804) according to the manufacturer’s instructions. Sample homogenization was performed using a Qiagen Vortex adapter coupled to a Vortex Genie 2 for 20 min at maximum speed. Initial deoxyribonucleic acid (DNA) quantification was performed on a NanoDrop (Thermo Scientific, Leicestershire, U.K.) and DNA concentration was adjusted to within the desired mass range for sequencing and plated on a 96 well, low profile, skirted polymerase chain reaction (PCR) plate (4titude, Surrey, U.K.) for sequencing submission. For bacterial 16S rRNA gene amplicon sequencing, the variable V3 and V4 regions of the 16S rRNA gene were amplified from genomic DNA using the primers (standard IUPAC nucleotide nomenclature): Forward Primer = 5' TCGTCGGCAGCGTCAGATGTGTATAAGAGACAGCCTACGGGNGGCWGCAGTCGTCGGCAGCGTCAGATGTGTATAAGAGACAGCCTACGGGNGGCWGCAG, Reverse Primer = 5’ GTCTCGTGGGCTCGGAGATGTGTATAAGAGACAGGACTACHVGGGTATCTAATCC.

The amplicons were then attached with indices and Illumina sequencing adapters using the Nextera XT index kit. The 16S amplicon libraries were pooled and sequenced in an Illumina MiSeq v3 flowcell as 300 paired-end reads. Library preparation and sequencing was performed at the Oxford Genomics Centre.

### Sequence processing

Quality of raw sequencing data was assessed using FastQC (v0.11.07). Following quality assessment, downstream processing was performed using dada2 (PMID:27214047) which was implemented in pipeline_dada2.py from the OCMS_16S repository (https://github.com/OxfordCMS/OCMS_16S). Given the drop in quality at ~ 200 bp, we decided to proceed with the analysis using the first read in the pair (V3 region). The filterAndTrim function in dada2 was used to truncate the reads to 200 bp. Retained primer sequences were removed from reads (17 bp) which resulted in final read lengths of 183 bp. Error learning, de-replication and sample inference were performed using dada2 with default parameters. Taxonomy was assigned to amplicon sequence variants (ASVs) using the assignTaxonomy function in dada2 and the Genome Taxonomy Database (GTDB) training data (https://zenodo.org/record/2541239/files/GTDB_bac-arc_ssu_r86.fa.gz). ASVs were retained if they made up at least 0.001% of the data set total read count in at least 5% of samples. ASVs were aggregated at either the genus or family level, depending on the analysis conducted.

### Microbiome analysis and statistics

Batch effects, potentially introduced by the veterinary center from which cats were recruited or by DNA extraction batch, were assessed using PERMANOVA on Bray–Curtis distances calculated from relative abundance of family-level taxa. This revealed that extraction batch did not have any significance, while the veterinary center did have a significant batch effect (data not shown). ComBat-Seq was used to correct for this batch effect, and batch-corrected counts were used in all subsequent analyses^26^.

α-Diversity was measured using Shannon’s diversity index and richness and β-diversity was measured using Bray–Curtis dissimilarity distance, which assess sample dissimilarity in a pairwise manner. Shannon’s diversity index, richness, and Bray–Curtis dissimilarity were calculated at the genus and family levels, using the vegan R package^27^. All pairwise significance testing was conducted using Wilcoxon Rank Sum test, with p-values adjusted by the Benjamini Hochberg (BH) method, and a significance threshold set at p-adjusted ≤ 0.05.

Microbial profiles were analyzed at the genus level, and aggregated counts were normalized using total sum-scaling and transformed by log_10_. Normalized, transformed, genus-level microbiome profiles were fitted to linear fixed effects modeling, with health status (healthy control vs. CE) as a fixed effect when comparing control and CE cats at baseline. When assessing the effect of diet in CE cats, a linear mixed effects model was used, where fixed effects were diet (pre vs. post), diet response (non-responder vs. responder), and diet-response interaction (change in non-responders vs. change in responders). Individual cat participants were included in the model as a random effect. All linear modeling were performed using Maaslin2^28^. Significance was determined using BH-adjusted p-value (q ≤ 0.1).

Microbiome profiles were converted into presence-absence data to assess the prevalence of each genus across groups. Contingency tables were created for each genus to assess the frequency observed in each group condition. Significance of contingency tables were assessed by Fisher’s Exact Test to estimate the probability of observing prevalence deviating from a perfectly even distribution across groups. For example, in control vs. CE, the null hypothesis was observation of 50% prevalence in both control and CE groups. Significance was determined using BH-adjusted p-value (q ≤ 0.1).

When assessing associations between clinical severity of CE and microbiome, FCEAI (1–9) and diet response (non-responder vs. responder) and FCEAI-response interaction (severity of non-responders vs. severity of responders) were used as fixed effects.

### Logistic regression analysis

Univariable binary logistic regression models were used to evaluate: 1. Presence or absence of fecal *Clostridium* in cats with CE and 2. Response or non-response to a hydrolyzed protein diet in cats with CE to determine if there were any associations with clinical measures. The clinical measures assessed were: referral center, signalment, duration and nature of signs, body condition score, laboratory parameters (serum albumin, cobalamin and folate concentrations and alanine aminotransferase (ALT) and alkaline phosphatase (ALP) activities), results from abdominal imaging and intestinal histopathology if available and FCEAI. Variables associated with the presence of fecal *Clostridium* or response to a hydrolyzed protein diet with *P* < 0.2 in simple logistic regression were subsequently entered into multivariable analyses. In the multivariable regression models, analyses were performed in a backward stepwise manner. All variables were initially included and the variable with the highest p-value was removed until all remaining variables had a *P* < 0.05. Analyses were performed using a computer software package (IBM SPSS Statistics Version 23).

### Ethical approval and consent to participate

The University of Bristol and Royal Veterinary College granted ethical approval for the study (VIN/14/017 and URN 2018 1837-3, respectively).

## Results

Cats with confirmed or suspected CE (*n* = 42) were prospectively enrolled from 2 referral veterinary hospitals in the U.K. Twenty-five cases were enrolled from the University of Bristol and 17 from the RVC. Three cases were excluded, as naturally voided feces could not be collected during hospitalization. The fecal microbiota were characterized in samples collected from the time of diagnostic investigations (*n* = 39) and after receiving a minimum of 6 weeks of the same commercial hydrolyzed protein diet (*n* = 25) in cats with CE and in control cats (*n* = 14).

Three cats in the CE group were subsequently excluded due to consequent diagnosis of small cell lymphoma (*n* = 2) or feline infectious peritonitis (*n* = 1). Thirty-six cats with suspected or confirmed CE were included in the study: 24 neutered male, 1 intact female and 11 neutered female. The age of the cats ranged from 0.8 years to 19 years, with a median age of 7 years. Eighteen cats were domestic shorthair, 5 domestic longhair, 4 Bengal, 3 Ragdoll, 2 Siamese and 1 each of the following breeds: Maine Coon, Abyssinian, Burmese and British Shorthair. Body condition score ranged from 2/9 to 9/9, with a median of 5/9.

The duration of GI signs for all cats in the CE group ranged between 0.5 months to 142 months (median, 6 months).

The control cats consisted of 14 staff owned cats: 10 male neutered, 3 female neutered and 1 female intact. The age of the cats ranged from 1 to 15 years with a median of 7 years. Breeds included 5 Domestic shorthair, 5 Domestic longhair and one each of the following breeds: British shorthair, Maine Coon, Russian Blue and Bengal. No control cats had a previous or current history of GI signs.

### Histologic diagnosis

Ten cats had intestinal biopsies performed, which found lymphoplasmacytic enteritis in 3 cats, lymphoplasmacytic and eosinophilic enteritis in 2 cats, lymphoplasmacytic and neutrophilic enteritis in 2 cats, mixed enteritis in 1 cat, lymphoplasmacytic and neutrophilic colitis in 1 cat and no abnormalities in 1 cat.

### FCEAI

The median FCEAI for all cats was 4, with a range of 1–9. For those cats that did not have endoscopy performed, a score of 0 was given for the variable endoscopic lesions^24^.

### Response to therapeutic hydrolyzed protein diet

Three cats did not eat the diet and were excluded from responder/non-responder analysis. Fifteen cats (45%) had full remission of signs (responders) and 18 cats (55%) had partial/no response (non-responders) after 6 weeks of feeding.

### Microbial communities: CE versus controls

**Figure 1:**
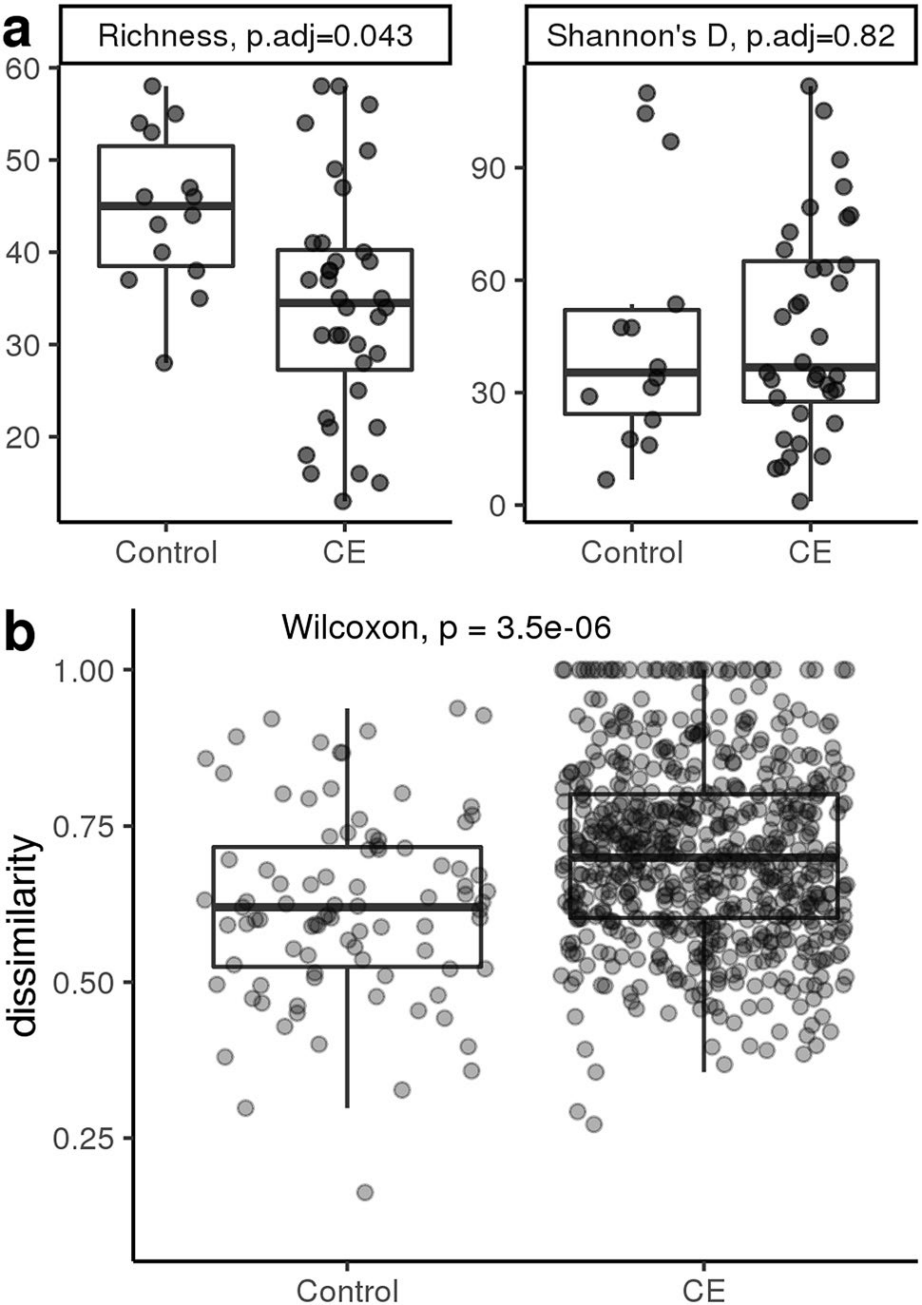
Diversity measures of control and chronic enteropathy (CE) cats at the genus level. (**a**) Genus richness (left panel) is significantly lower in CE cats at time of diagnosis as compared to control cats. Diversity as measured by Shannon’s Diversity Index (right panel) is not significant between CE and control cats at time of diagnosis. (**b**) Sample dissimilarity in control and CE cats shows that CE cats tend to be more dissimilar from other CE cats as compared to the dissimilarity of control cats from other cats in the same group.

The fecal microbiome of cats with CE showed decreased α-diversity in terms of genus richness (*P* = 0.04) and increased β-diversity in terms of Bray–Curtis Dissimilarity (*P* < 0.001) compared to control cats (Fig. 1a and b).

**Figure 2:**
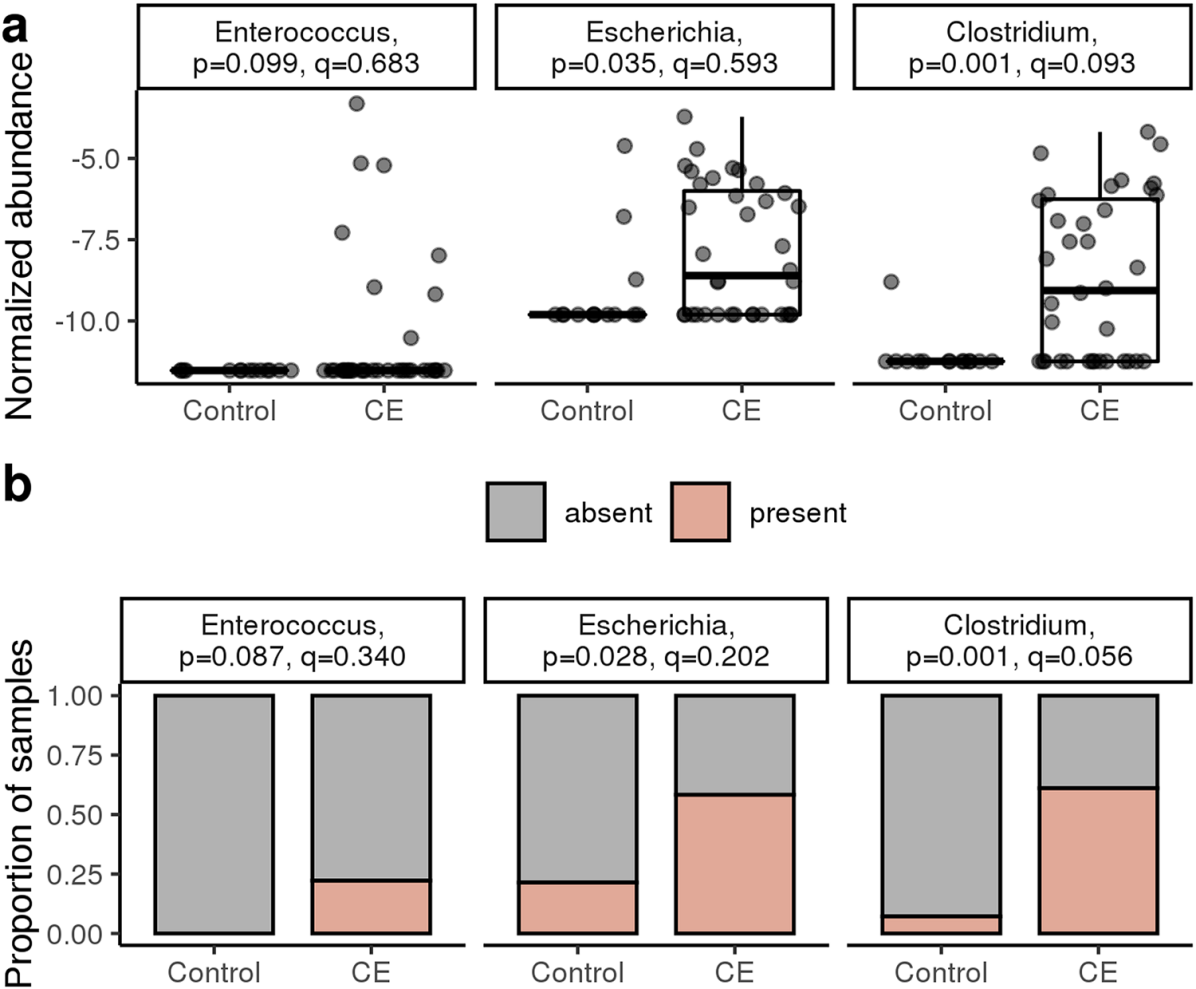
Comparison of selected bacterial genera associated with chronic enteropathy (CE) in dogs and cats and humans with inflammatory bowel disease in the existing literature (*Enterococcus, Escherichia* and *Clostridium)* in cats with CE as compared to control cats. Although, all 3 selected genera are over-represented in cats with CE as compared to control cats, only Clostridium was statistically significant (q < 0.1). (**a**) Normalized abundance of *Enterococcus*, *Escherichia*, and *Clostridium* in control and CE cats at time of diagnosis. Statistical testing performed on linear fixed model, with significance at q < 0.1. (**b**) Contingency analysis showing prevalence of a given genus in control and CE cats.

Based on the linear model and contingency analyses, most genera are over-represented in control cats as compared to CE cats (Supplementary Fig. 1). This is consistent with the α-diversity and sample dissimilarity results described above. Bacterial genera associated with CE in dogs and cats and humans with IBD in the existing literature (*Enterococcus, Escherichia* and *Clostridium*) were also over-represented in the cats with CE as compared to control cats, albeit only *Clostridium* was statistically significant (adjusted p-value < 0.1, Table 1 and Fig. 2a and b).

**Table 1:** Comparison between fecal microbiota in cats with chronic enteropathy and control cats with no gastrointestinal signs. *Clostridium* was the only genus that was statistically significant after adjusting for multiple testing.

tax_level	featureID	coef	stderr	qval
Genus	Clostridium	2.3826	0.6741	0.0933

### Effect of diet on the fecal microbiota of cats with CE

**Figure 3:**
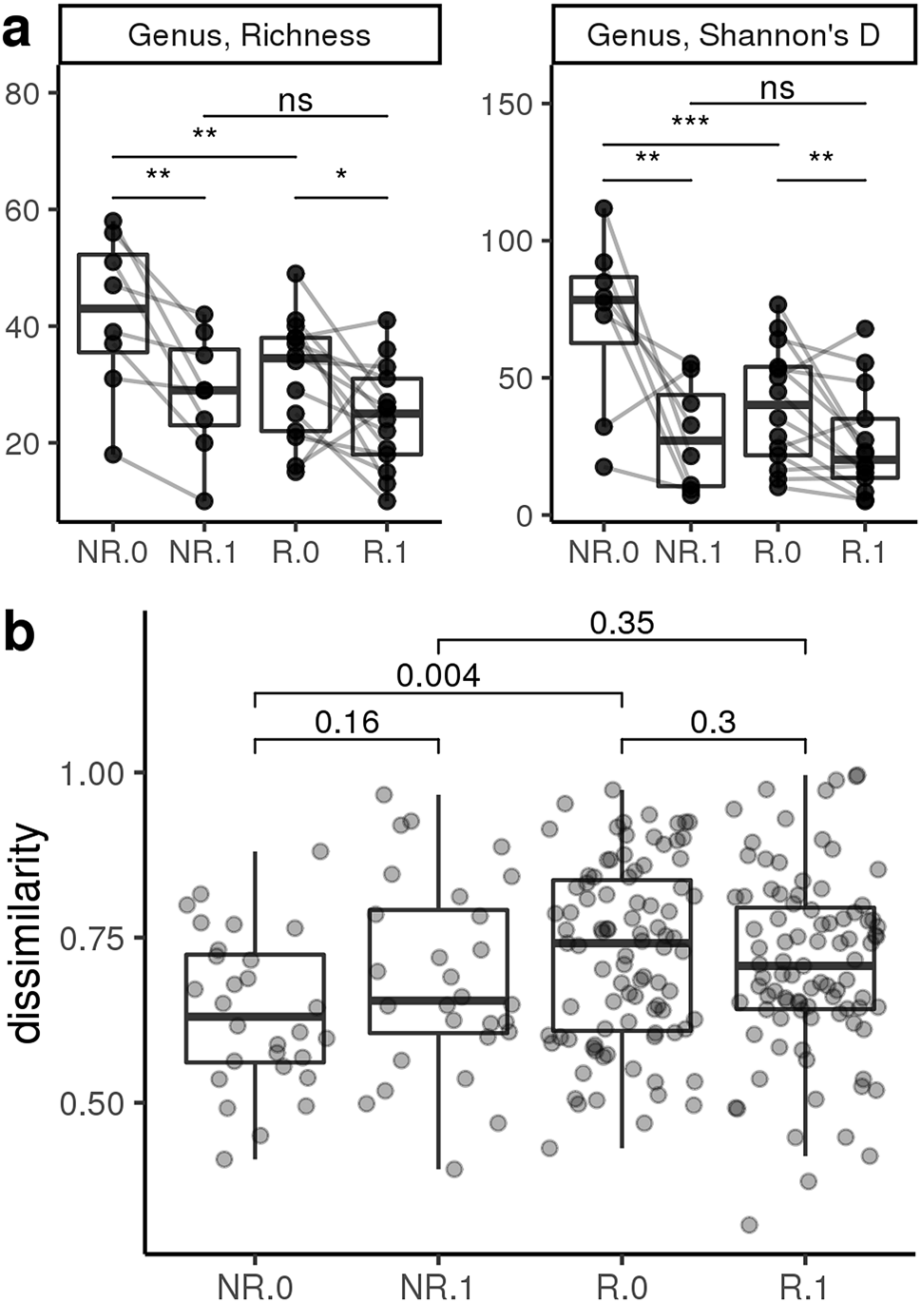
Diversity measures at the genus level of chronic enteropathy (CE) cats at time of diagnosis and post dietary intervention. NR = non-responder, R = responder, 0 = time of diagnosis, 1 = post dietary intervention (**a**) Genus richness (left panel) and diversity measured by Shannon’s Diversity Index (right panel) are lower in responders than non-responders at time of diagnosis. Dietary intervention resulted in decreased richness and diversity in both groups such that post-treatment diversity measures are not significantly different between responders and non-responders. Adjusted p-value < 0.0001 represented with ***. Adjusted p-value < 0.001 represented with **. Adjusted p-value < 0.01 represented with *. Adjusted p-value > 0.05 represented with ns. (**b**) Sample dissimilarity shows that responders have greater within-group dissimilarity than non-responders at the time of diagnosis. Following dietary intervention, non-responders and responders have equivalent within-group dissimilarities.

**Figure 4:**
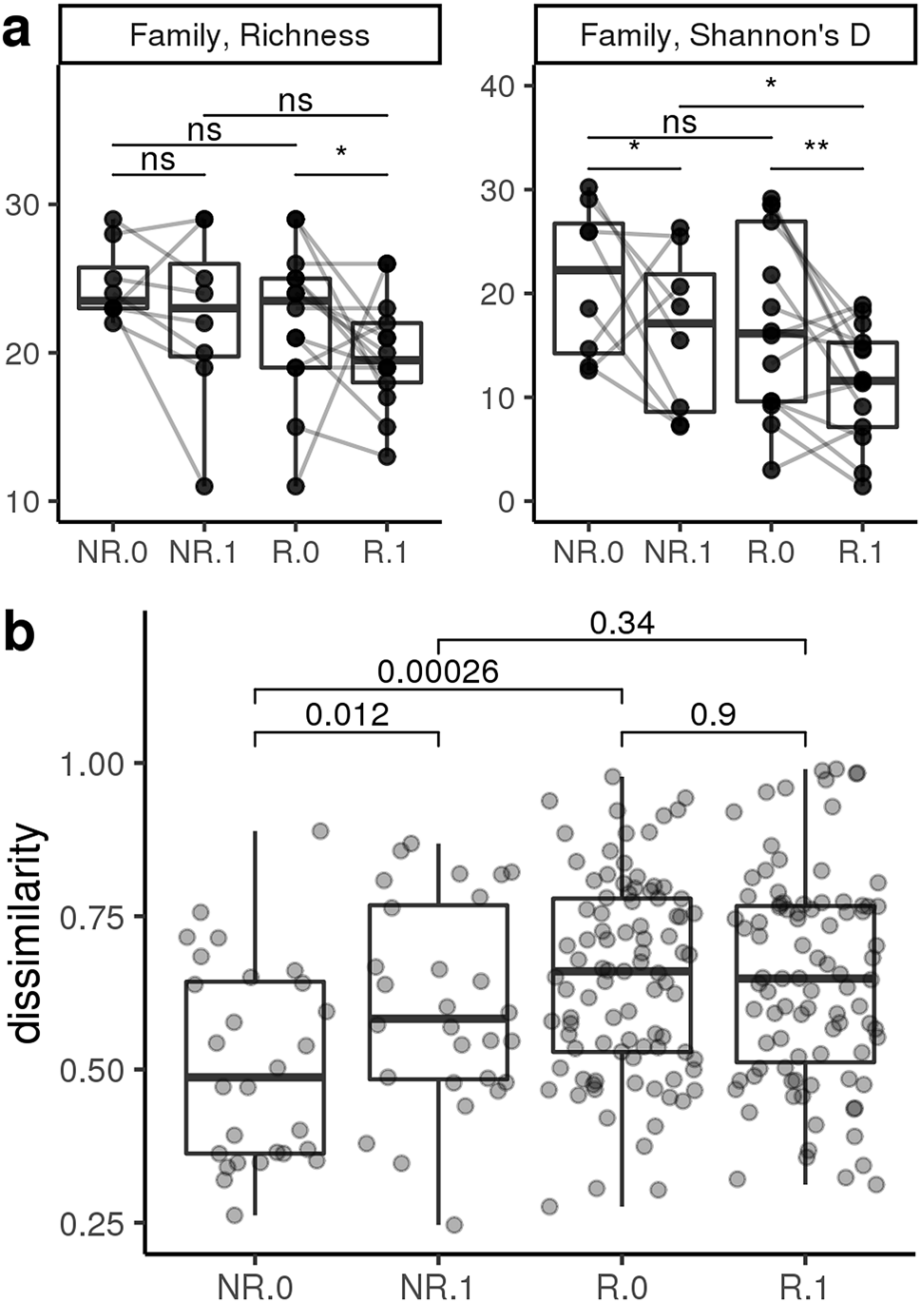
Diversity measures at the family level of chronic enteropathy (CE) cats at time of diagnosis and post dietary intervention. NR = non-responder, R = responder, 0 = time of diagnosis, 1 = post dietary intervention (**a**) Family richness (left panel) is decreased in responders but remains unchanged in non-responders. Diversity as measured by Shannon’s Diversity Index (right panel) is decreased after dietary treatment, regardless of clinical response to the dietary intervention. Adjusted p-value < 0.0001 represented with ***. Adjusted p-value < 0.001 represented with **. Adjusted p-value < 0.01 represented with *. Adjusted *P*-value > 0.05 represented with ns. (**b**) Sample dissimilarity shows that non-responders become more dissimilar after dietary intervention. On the other hand, responders maintain the same level of sample dissimilarity after dietary intervention, even though responders are more heterogeneous in β-diversity than non-responders at the time of diagnosis.

Shannon’s Diversity Index for α-diversity decreased over time in both responders and non-responders (*P* < 0.05) at both the genus and family level (Figs. 3 and 4). At the genus level, β-diversity was different between the responders and non-responders at diagnosis (*P* = 0.004, Fig. 3), but not between the 2 groups after dietary intervention (*P* = 0.35, Fig. 3). However, at the family level, non-responders became increasingly dissimilar after dietary intervention (β-diversity, *P* = 0.012, Fig. 4).

**Figure 5:**
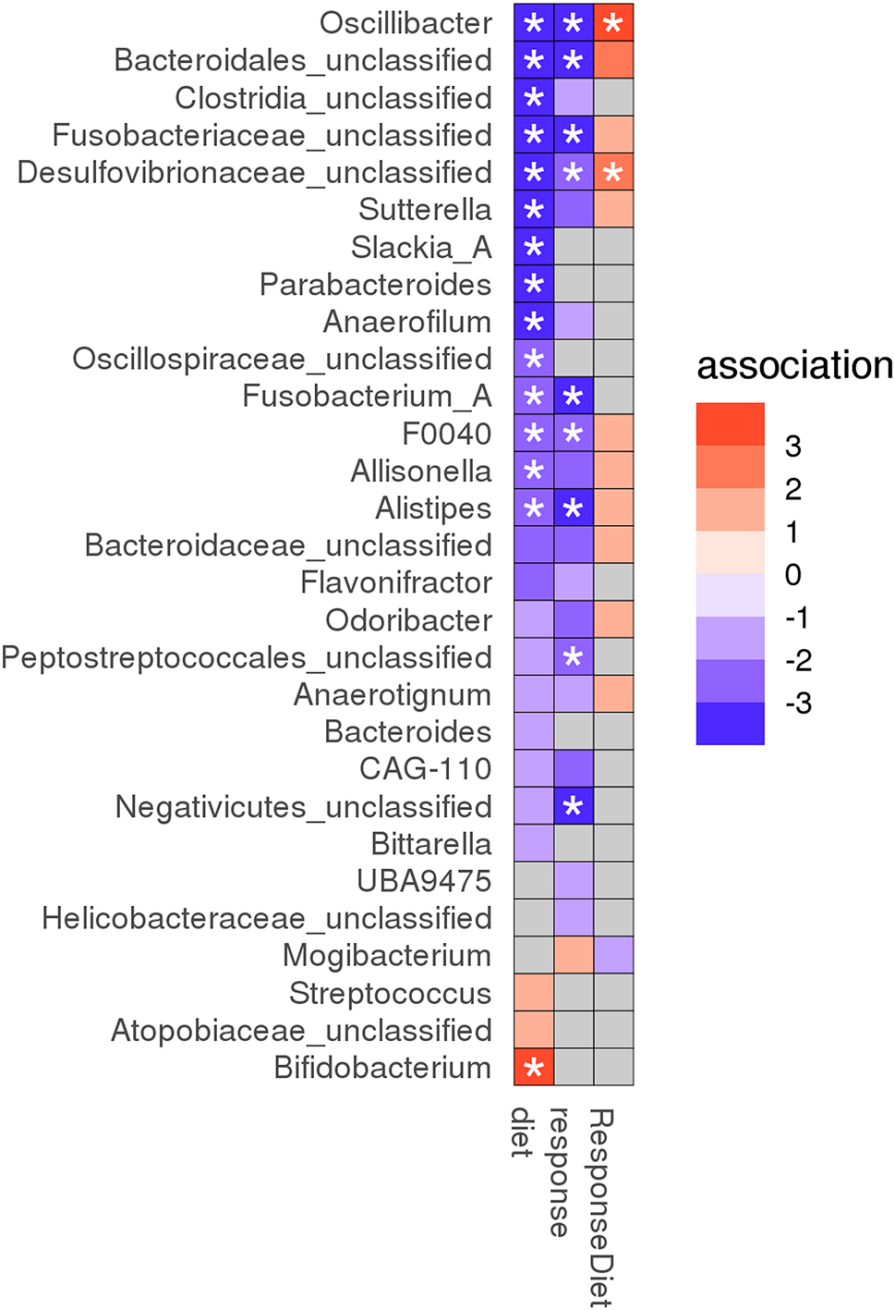
Dietary response analysis in cats with chronic enteropathy (CE). Significance of feature coefficients in linear model where fixed effects are diet, response, and interaction of the two (ResponseDiet). q < 0.1 shown with a star. *P*-values > 0.05 are colored in grey. Association is -log(q-value)*sign(coefficient).

**Figure 6:**
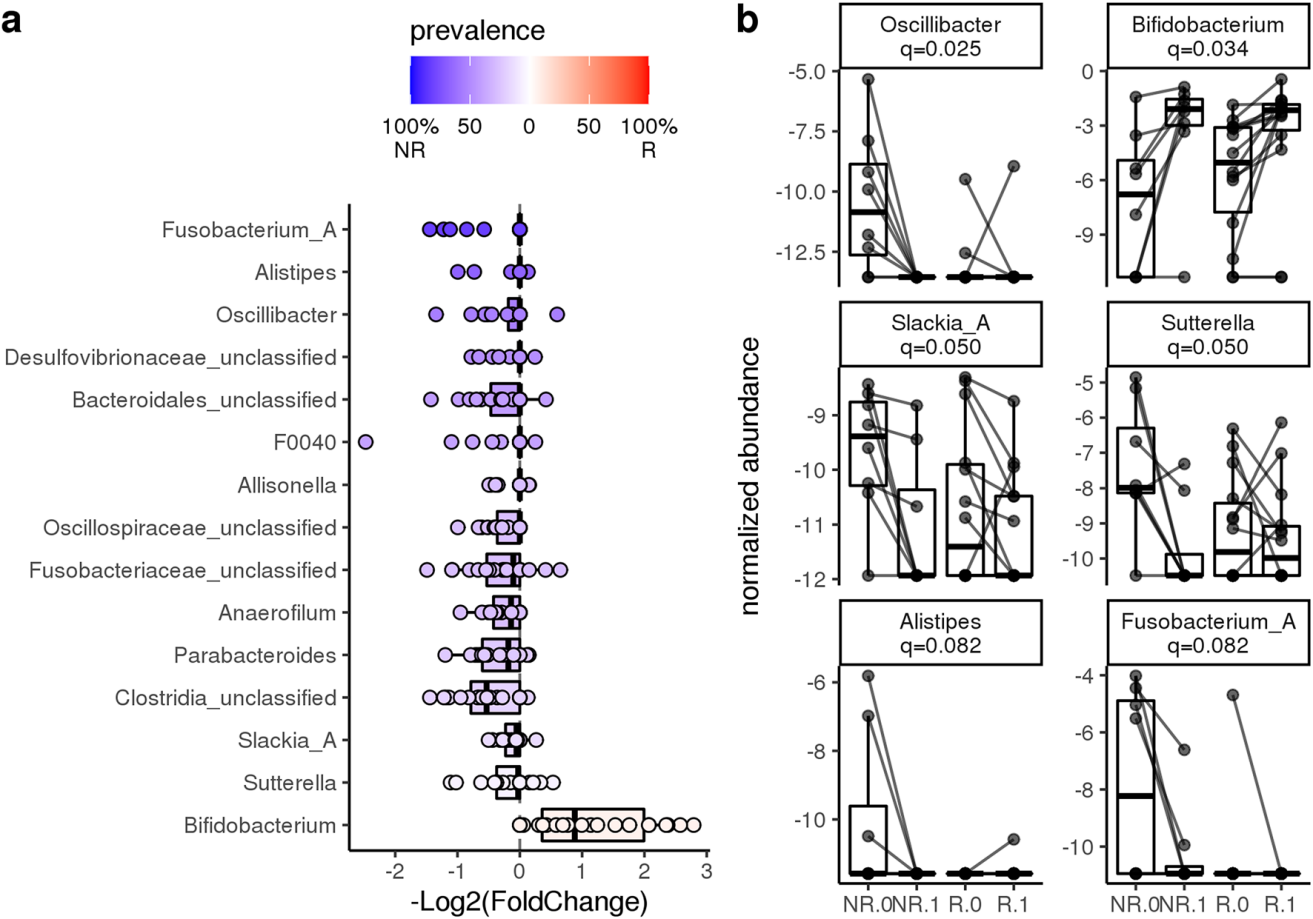
The effect of diet in cats with chronic enteropathy (CE). The majority of the significant features identified by the model are due to the effect of diet. In general, the abundance of bacteria decreases with diet. In particular, the bacteria most significantly affected by diet (q < 0.1) were more frequently observed in non-responders (dark purple circles in panel **A**). *Bifidobacterium* was the only significant genus that increased in abundance post-diet, an effect that was observed in both responders and non-responders.

Fecal bacterial genera that were significantly associated with diet alone, response alone, and interaction of diet with response are shown in Table 2 and summarized in Fig. 5. The majority of the significant features identified by the model are due to the effect of the diet alone (Table 2 and Fig. 5). In general, the abundance of bacteria decreases with diet. In particular, the bacteria most significantly affected by diet (adjusted p-value < 0.1) were more frequently observed in non-responders (dark purple circles in panel a of Fig. 6). For example, *Oscillibacter*, *Sutterella* and *Fusobacterium* were all present in higher frequency in non-responders at baseline as compared to responders (Fig. 6, panel b).

**Table 2:** Dietary response analysis in cats with chronic enteropathy. Significance of feature coefficients in linear model where fixed effects are diet, response, and interaction of the two (ResponseDiet).

fixed_effect	tax_level	featureID	Coef.	stderr	qval
diet	Phylum	*Desulfobacterota*	−0.7171	0.1886	0.0168
diet	Phylum	*Fusobacteriota*	−1.8208	0.5451	0.0182
diet	Phylum	*Actinobacteriota*	0.6413	0.2527	0.0979
diet	Class	*Actinobacteria*	1.8197	0.5155	0.0173
diet	Class	*Desulfovibrionia*	−0.7171	0.1886	0.0173
diet	Class	*Fusobacteriia*	−1.8208	0.5451	0.0173
diet	Order	*Clostridia_unclassified*	−1.7101	0.3949	0.0210
diet	Order	*Desulfovibrionales*	−0.7171	0.1886	0.0210
diet	Order	*Actinomycetales*	1.8197	0.5155	0.0263
diet	Order	*Fusobacteriales*	−1.8208	0.5451	0.0263
diet	Order	*Betaproteobacteriales*	−1.2150	0.3957	0.0607
diet	Family	*Bacteroidales_unclassified*	−1.7966	0.4358	0.0203
diet	Family	*Clostridia_unclassified*	−1.7101	0.3949	0.0203
diet	Family	*Oscillospiraceae*	−1.9780	0.4854	0.0203
diet	Family	*Bifidobacteriaceae*	2.0469	0.5315	0.0210
diet	Family	*Desulfovibrionaceae*	−0.7171	0.18867	0.0210
diet	Family	*Fusobacteriaceae*	−1.8208	0.5451	0.0303
diet	Family	*Tannerellaceae*	−1.2485	0.3653	0.0372
diet	Family	*Burkholderiaceae*	−1.2150	0.3957	0.0601
diet	Family	*Dialisteraceae*	−0.5376	0.1875	0.0601
diet	Family	*Rikenellaceae*	−0.7252	0.2535	0.0601
diet	Genus	*Bacteroidales_unclassified*	−1.7966	0.4359	0.0248
diet	Genus	*Oscillibacter*	−1.5721	0.3889	0.0248
diet	Genus	*Clostridia_unclassified*	−1.7101	0.3949	0.0248
diet	Genus	*Bifidobacterium*	2.0469	0.5315	0.0343
diet	Genus	*Desulfovibrionaceae_unclassified*	−0.7171	0.1886	0.0343
diet	Genus	*Fusobacteriaceae_unclassified*	−1.3969	0.3968	0.0343
diet	Genus	*Anaerofilum*	−0.9192	0.2688	0.0497
diet	Genus	*Parabacteroides*	−1.2485	0.3654	0.0497
diet	Genus	*Slackia_A*	−0.7206	0.2081	0.0497
diet	Genus	*Sutterella*	−1.1930	0.3356	0.0497
diet	Genus	*Alistipes*	−0.7252	0.2535	0.0822
diet	Genus	*Allisonella*	−0.5376	0.1875	0.0822
diet	Genus	*F0040*	−1.5446	0.5108	0.0822
diet	Genus	*Fusobacterium_A*	−1.2287	0.3925	0.0822
diet	Genus	*Oscillospiraceae_unclassified*	−0.6801	0.2249	0.0822
response	Phylum	*Fusobacteriota*	−3.5201	0.9553	0.0168
response	Phylum	*Desulfobacterota*	−1.5313	0.5364	0.0473
response	Class	*Fusobacteriia*	−3.5201	0.9553	0.0173
response	Class	*Desulfovibrionia*	−1.5313	0.5364	0.0433
response	Order	*Fusobacteriales*	−3.5201	0.9553	0.0210
response	Order	*Negativicutes_unclassified*	−1.8704	0.5258	0.0210
response	Order	*Desulfovibrionales*	−1.5313	0.5364	0.0657
response	Family	*Bacteroidales_unclassified*	−2.8610	0.7638	0.0203
response	Family	*Fusobacteriaceae*	−3.5201	0.9553	0.0203
response	Family	*Negativicutes_unclassified*	−1.8704	0.5258	0.0210
response	Family	*Rikenellaceae*	−1.4338	0.4443	0.0372
response	Family	*Peptostreptococcales_unclassified*	−0.9831	0.3128	0.0395
response	Family	*Oscillospiraceae*	−2.5881	0.9062	0.0601
response	Family	*Desulfovibrionaceae*	−1.5313	0.5364	0.0658
response	Family	*Dialisteraceae*	−0.8983	0.3285	0.0732
response	Family	*Marinifilaceae*	−1.7801	0.6792	0.0890
response	Genus	*Oscillibacter*	−2.7462	0.6816	0.0248
response	Genus	*Bacteroidales_unclassified*	−2.8610	0.7638	0.0291
response	Genus	*Fusobacteriaceae_unclassified*	−2.6029	0.6954	0.0291
response	Genus	*Negativicutes_unclassified*	−1.8703	0.5258	0.0343
response	Genus	*Alistipes*	−1.4338	0.4443	0.0497
response	Genus	*Fusobacterium_A*	−2.6494	0.8007	0.0497
response	Genus	*Peptostreptococcales_unclassified*	−0.9831	0.3128	0.0537
response	Genus	*F0040*	−2.7156	0.9365	0.0822
response	Genus	*Desulfovibrionaceae_unclassified*	−1.5313	0.5364	0.0928
ResponseDiet	Phylum	*Desulfobacterota*	0.6695	0.2203	0.0473
ResponseDiet	Class	*Desulfovibrionia*	0.6695	0.2203	0.0427
ResponseDiet	Order	*Desulfovibrionales*	0.6695	0.2203	0.0607
ResponseDiet	Family	*Desulfovibrionaceae*	0.66951	0.2203	0.060
ResponseDiet	Family	*Bacteroidales_unclassified*	1.3319	0.5089	0.0890
ResponseDiet	Genus	*Oscillibacter*	1.4489	0.4542	0.0497
ResponseDiet	Genus	*Desulfovibrionaceae_unclassified*	0.6695	0.2203	0.0822

*Bifidobacterium* was the only significant genus that increased in abundance post-diet, an effect that was observed in both responders and non-responders (Table 2, Figs. 5 and 6).

**Figure 7:**
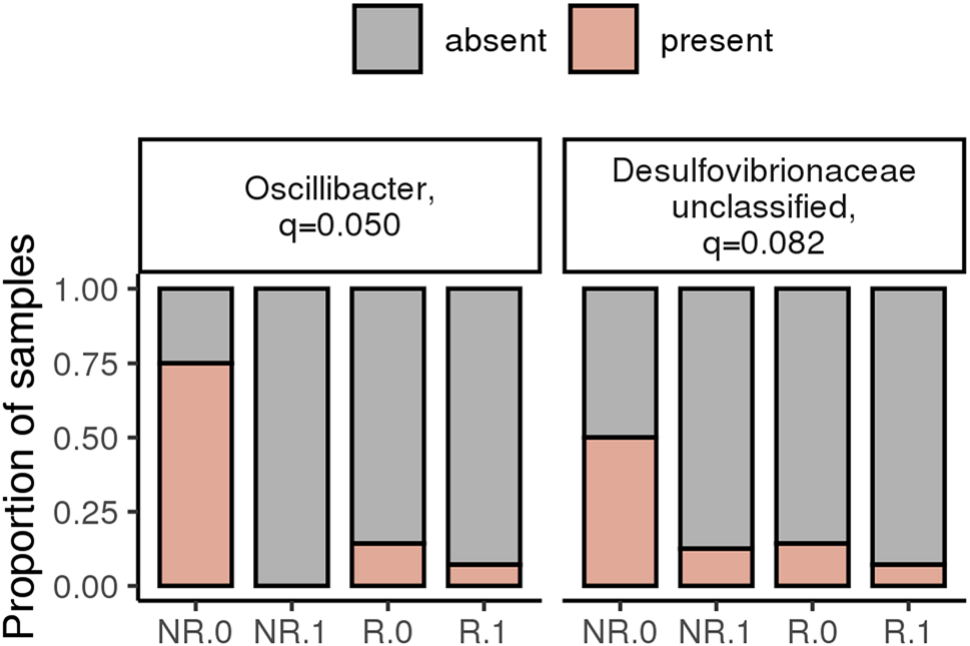
Differing effects of diet for responders and non-responders in cats with chronic enteropathy (CE). There are a limited number of significant interaction terms, where the only significant genera are *Oscillibacter* and *Desulfoviberionaceae_unclassified*. Both are most prevalent in non-responders at baseline and are rarely observed post diet in both non-responders and responders.

The interaction term describes if responders respond differently to the diet than non-responders. As seen in Fig. 5, there are a limited number of significant interaction terms, where the only significant genera are *Oscillibacter* and *Desulfoviberionaceae_unclassified*. Both are most abundant in non-responders at baseline and are rarely observed post diet in both non-responders and responders (Fig. 7).

### Effect of clinical severity (FCEAI) on fecal microbiome

Linear fixed effect modeling was assessed at every taxonomic level where fixed effects are response, clinical severity (FCEAI), and interaction of two variables (FCEAI_Response). No terms were significant at the Genus level. However, the following taxa were significant in either the clinical severity or the response_severity coefficients: Campylobacterota, Campylobacteria, Tannerellaceae (Supplementary Table 1 and Supplementary Fig. 2). Although these taxa were significant, their average abundance was < 0.5%.

The families Tannerellaceae (q = 0.07) and Helicobacteraceae (q = 0.15) both decreased in abundance with increasing severity in non-responders (Supplementary Table 1). On the other hand, abundance in responders is agnostic to CE severity, as either because it is absent or because levels remain consistent (Supplementary Fig. 3). Even though the response-severity interaction for Helicobacteraceae was not statistically significant, the trend was significantly observed at its class and phylum levels, Campylobacteria (q = 0.061) and Campylobacterota (q = 0.056), respectively. Similarly, Tannerellaceae’s response-severity interaction was recapitulated at the class (Bacteroidia) and phylum (Bacteroidota) level (Supplementary Table 2).

### Logistic regression analysis for presence or absence of fecal Clostridium species in CE

Univariable logistic regression models showed no significant association between the presence of fecal *Clostridium* in cats with CE and referral center, signalment, duration and nature of signs, body condition score, laboratory parameters (albumin, cobalamin, folate, ALT and ALP), or results from abdominal imaging and intestinal histopathology and FCEAI (*P* > 0.338).

### Logistic regression analysis for response or non-response to therapeutic hydrolyzed protein diet

Univariable logistic regression models showed no significant association between response to a hydrolyzed protein diet in cats with CE and referral center, signalment, duration and nature of signs, body condition score, laboratory parameters (albumin, cobalamin, folate, ALT and ALP), or results from imaging and intestinal histopathology and FCEAI (*P* > 0.117).

## Discussion

This study identified that at the genus level the fecal microbiota of cats with CE had a lower α-diversity and higher β-diversity than healthy control cats. This is consistent with previous reports comparing the fecal microbiota of cats with CE and clinically healthy cats^11^ and in dogs with CE^6–8^ and humans with IBD^3–5^. Although, it is unknown if these changes are the cause or consequence of the intestinal inflammation, studies in rodent models of IBD have shown that the microbiome is essential for the initiation and perpetuation of mucosal inflammation, as intestinal changes are absent when these models are raised in a germ-free environment^29,30^. Additionally, intestinal inflammation can be elicited in healthy rodents following transfer of the microbiome from rodents with IBD^31,32^.

In this study, the genus *Clostridium* was significantly higher in the feces of cats with CE compared to those of control cats at diagnosis. Several studies in human IBD have documented decreased abundance of *Clostridium*^33^, with one study documenting a negative correlation with disease severity^34^. A systematic review showed that people with active IBD had lower abundance of *Clostridium coccoides* and *Clostridium leptum*^35^, whereas *Clostridium difficile* has been shown to be prevalent in patients with ulcerative colitis^36,37^. Similar to humans with IBD, dogs with CE have been shown to have decreased species of *Clostridium* (e.g. *C. hiranosis*) but an increase in other species (e.g. *C. perfringens*)^7,38^. In this study, although *Clostridium* was more abundant in cats with CE, further studies are needed to determine the species involved, as a previous study showed that mucosally associated *Clostridium* spp. in cats with CE correlated with abnormalities in mucosal architecture, upregulation of cytokine mRNA and the number of clinical signs exhibited by the cats^39^.

The bacterial genera, *Enterococcus* and *Escherichia* have been associated with CE in dogs and cats and with IBD in humans^5,11,21,39–41^. These two genera were also over-represented in cats with CE as compared to control cats in our study. Although, neither reached significance after multiple comparison testing, the comparatively smaller number of cats in our control group may have reduced the power of detecting a significant difference. Therefore, studies incorporating a larger number of cats in both groups are needed to confirm or refute the role of *Enterococcus* and *Escherichia* in the pathogenesis of feline CE*.*

Interestingly, we noted a bimodal distribution of our dataset with regards to the fecal microbiota. We observed the feline microbiome to be sparse, such that microbiome profiles tended to be binary, where genera could be described as either present or absent. This type of distribution is important for the research community to be aware of, so they can account for the sparse nature of the feline fecal microbiome profiles. Analyses that account for the prevalence of genera, such as contingency testing, can be used to complement linear microbiome modeling methods when assessing differential abundances.

The rate of clinical remission achieved with a hydrolyzed protein diet in our study was only 45%. Alpha-diversity decreased over time in both dietary responders and non-responders, with the abundance of bacteria decreasing with the hydrolyzed protein diet. In particular, the bacteria most significantly affected by diet were more frequently observed in cats in the non-responder group. Therefore, despite a reduction in α-diversity and frequently present bacteria in the non-responder group, these cats still had persistent GI signs. This suggests that these cats likely require a different dietary strategy or medication to help improve their clinical signs, due to the differing etiopathogenesis of their disease. This is also exemplified by the fact that the β-diversity at the family level became increasingly dissimilar after dietary intervention in the non-responders. Therefore, dietary responders metabolize the diet similarly due to their sustained β-diversity following dietary intervention, whereas cats in the non-responder group likely represent a different group of cats with regards to the etiopathogenesis of their CE that requires a different therapeutic dietary strategy or medication for clinical remission. Further investigation would be needed to determine whether cats in the non-responder group are still food-responsive, potentially responding to another dietary strategy, such as limited-ingredient novel protein diet, or are steroid-responsive. If these cats are steroid-responsive they may have a specific aberration in their immune system, which results in a less tightly controlled microbiota, allowing for a more diverse response following dietary intervention. The gastrointestinal mucosal immune system is vital to homeostasis with regards to the microbiota and is home to a unique set of complex immunoregulatory mechanisms that help provide an appropriate immune response to commensal microorganisms^42^. Therefore, disruption of this highly complex network of immunological pathways may lead to chronic inflammation, which may alter their microbiological response to diet. Consequently, these cats may require additional therapy, such as glucocorticoids, to reduce the aberrant immune response, allowing them to enter clinical remission. Identifying the underlying reason for the increased β-diversity in non-responders following intervention with a hydrolyzed protein diet will help to advance our knowledge of the etiopathogenesis of the different sub-types of this disease and therefore guide the use of the most effective treatment for these cats.

Fecal *Bifidobacterium* was higher following dietary intervention in both the responder and non-responder groups. A previous study identified decreased *Bifidobacterium* species in the feces of cats with CE^43^. Therefore, an increase in this genus following dietary intervention may have helped the cats in the responder group enter remission. *Bifidobacterium* is considered beneficial in the gut due to its role in fermentation of fiber resulting in the production of the short-chain fatty acid butyrate. Butyrate is a major fuel source for colonocytes^44^ and helps to keep the intestine anaerobic, thereby inhibiting the growth of pathogenic bacteria. Butyrate also has direct effects on intestinal barrier function and the intestinal immune system^45–47^. Previous studies utilizing fructooligosaccharide (FOS) supplementation in cats demonstrated that these cats had higher levels of *Bifidobacteria* and increased levels of fecal butyrate^48^. The hydrolyzed protein diet utilized in our study contains FOS and is, therefore, the likely source for the increase in this bacterial genus. However, the reason for non-response in some cats following intervention with this diet, despite an increase in *Bifidobacterium* requires further investigation. One possible reason could be the predominant location of inflammation within the GI tract, with large intestinal disease more likely responding to the beneficial effects of FOS. Therefore, further studies correlating fecal microbiota changes with localization of inflammation within the GI tract will help to tease out the phenotype of CE cats that are more likely to benefit from a probiotic or higher dietary fiber strategy.

*Oscillibacter* and *Desulfoviberionaceae_unclassified* were most abundant in non-responders at diagnosis compared to responders but were rarely observed post diet in either group. In a mouse model of ulcerative colitis, *Oscillibacter* abundance was significantly positively associated with the pro-inflammatory cytokines interleukin (IL)-6 and IL-1 β and pathological scores^49^. This same genus was shown to increase transepithelial resistance of the proximal colon and reduce mRNA expression of tight junction protein, zona occludens 1, in the colon of obese mice^50^. This suggests that the increased abundance of this genus increases inflammation and compromises intestinal barrier function in dietary non-responders at diagnosis and may explain why additional treatment may be warranted to reduce GI signs in these cats. However, the hydrolyzed diet did reduce the abundance of this genus in the feces of non-responders, which suggests that other mechanisms may need to be targeted in addition to resolve GI signs in this cohort of cats. However, further studies assessing the effect of diet on immunological and barrier function in these cats will help to determine the mechanism of action of these diets and whether on a sub-clinical level their continued use is warranted in these cats, together with additional treatment.

*Desulfovibrio *spp., which are producers of toxic sulfides are significantly more abundant in cats with CE and humans with IBD^43,51^. Hydrogen sulfide is a highly toxic metabolic end product of *Desulfovibrio *spp. and is thought to inhibit oxidation of butyrate^52^. Indeed, in vitro, hydrogen sulfide selectively inhibited oxidation of butyrate by colonocytes, which led to lesions associated with colitis^53^. Also, *Desulfovibrio *spp., may produce immunogenic compounds that may result in an immune response in susceptible individuals^54^. Therefore, their increased abundance in non-responders at diagnosis may reflect their role in the pathogenesis of CE in these cats. The abundance of this genus did decrease following dietary intervention in non-responders. Interestingly, short-chain fatty acids produced by *Bifidobacteria* may inhibit the growth of *Desulfovibrio *spp. due to their effect on luminal pH, the direct inhibitory effect of short-chain fatty acids or production of antimicrobial peptides targeted towards these bacteria^55^. Such inter-microbial interactions may be a participating mechanism for the decrease in abundance of the genus *Desulfoviberionaceae_unclassified* in our study. However, despite the decrease in this genus following dietary intervention, cats in the non-responder group did not enter clinical remission, again suggesting that additional changes in the microbiota or immune system of these cats are needed to bring about remission. Assessing the metabolome of cats in the non-responder group before and after dietary intervention and comparing this to responder cats will help to further highlight the pathways involved in the pathogenesis of the different subtypes of this disease and the mechanism of action of this dietary intervention in these two group of cats. Further studies are also needed to help determine whether the presence of *Oscillibacter* and *Desulfoviberionaceae_unclassified* at diagnosis of CE in cats helps to predict subsequent response to hydrolyzed protein dietary intervention.

Interestingly, non-responders had reduced levels of the significant taxa; Campylobacterota, Campylobacteria and Tannerellaceae with increasing FCEAI, but this trend was not seen in the responders. One possibility could be that more severe CE results in more differing ecological niches that are less favorable for the growth of those organisms. Therefore, microbiota and histopathological characterization from intestinal mucosal biopsy specimens from all responders and non-responders would give us better insight into whether they differ in their inflammatory landscape to explain why the two groups follow different severity vs. abundance trends.

Although our data suggests that a hydrolyzed protein diet is able to modify the microbiome, the diet may induce clinical remission via mechanisms independent of the microbiome and, therefore, the alterations in the microbiota seen in our study may be a consequence rather than the direct cause of remission. Further studies assessing how dietary therapy may help to improve remission rates in cats with CE, by assessing the metabolome are therefore needed, as this will help to guide evidence-based design of more efficacious therapeutic diets.

Our study had the following limitations: although cats in the control group did not have any reported GI signs, full physical examination and laboratory blood work was not performed to ensure subclinical GI disease was not present nor was a full history collected to rule out other diseases or current medications. The control cats did not receive dietary intervention with the hydrolysed protein diet and therefore, repeat fecal samples were not collected for comparisons. Acquiring this information could have helped to provide more context for the changes seen in our study and therefore should be addressed in future studies assessing the impact of dietary intervention on the fecal microbiome in cats. Similarly, our study did not include a control diet to help compare the specific effects of the hydrolysed diet against and as such future studies should also focus on a group of cats with chronic enteropathy consuming a control diet, such as a commercial therapeutic gastrointestinal highly digestible diet. In addition, the number of control cats in our study was relatively small and therefore future studies should focus on recruiting a larger population of control cats. Although, all owners were specifically instructed to feed the diet exclusively, which included the use of no treats or additional foods, flavored medication such as deworming medication or toothpaste was not specifically advised against. An owner completed daily diary to verify all oral intake would have helped to confirm exclusive feeding and should be considered for future studies. In addition, the indoor/outside status of the cat and access to additional foods outside and as part of a multicat household was not specifically taken into consideration. However, a recent study suggested that dogs with food-responsive enteropathy did not need to receive these diets exclusively for effectiveness at controlling clinical signs^56^.The fecal microbiota might not accurately represent the mucosa-associated microbiota^57^ and therefore assessing the latter might have helped to identify additional changes between the groups. Finally, not all cats had GI biopsies performed to confirm an underlying diagnosis of chronic inflammatory enteropathy. Although two cats were excluded due to alimentary small cell lymphoma (SCL) on subsequent GI biopsy, additional cats may have been excluded if they all had this procedure performed. Nevertheless, a recent study did not find any bacterial taxa that differed between cats with chronic inflammatory enteropathy and SCL and concluded that their findings might lend support to the hypothesis that these two diseases are not different but rather a continuum^11^.

## Conclusion

This study showed that the fecal microbiota of cats diagnosed with CE is significantly different from control cats without GI signs, and that these cats had similar changes to those described in human IBD. Despite the hydrolyzed protein diet reducing α-diversity in all cats with CE, this did not resolve GI signs in some cats. However, the microbiome of non-responders became increasingly dissimilar compared to diagnosis at the family level. Therefore, the microbiome may not be as tightly regulated by the immune system in cats with CE that are non-responders and therefore, may be the reason these cats require additional therapy for remission of GI signs. Whether *Oscillibacter* and *Desulfovibrionaceae_unclassified* are indicators of non-response to the diet at diagnosis requires further investigation. Further studies determining the metabolomic pathways for dietary responders and non-responders will enable the development of more efficacious therapeutic diets that might help all cats to attain remission with dietary therapy alone.

## Supplementary Information

Supplementary Information 1.

Supplementary Information 2.

Supplementary Information 3.

Supplementary Information 4.

## Data Availability

All data generated or analyzed during this study are included in this published article and its supplementary information files.

## Abbreviations

ALP: Alkaline phosphatase
ALT: Alanine aminotransferase
ASVs: Amplicon sequence variants
BH: Benjamini Hochberg
CE: Chronic enteropathy
DNA: Deoxyribonucleic acid
FCEAI: Feline chronic enteropathy activity index
FOS: Fructooligosaccharide
FRE: Food responsive enteropathy
GI: Gastrointestinal
GTDB: Genome Taxonomy Database
IBD: Inflammatory bowel disease
IL: Interleukin
NR: Non-responder
PCR: Polymerase chain reaction
PLI: Pancreatic lipase immunoreactivity
R: Responder
RVC: Royal Veterinary College
SCL: Small cell lymphoma
SRE: Steroid responsive enteropathy
T4: Thyroxine
TLI: Trypsin-like immunoreactivity
