# Supplementary figures and images for: The effect of a hydrolyzed protein diet on the fecal microbiota in cats with chronic enteropathy
Source: Sci Rep. 2022 Feb 17;12:2746. doi: 10.1038/s41598-022-06576-y (PMC8854717; doi:10.1038/s41598-022-06576-y)

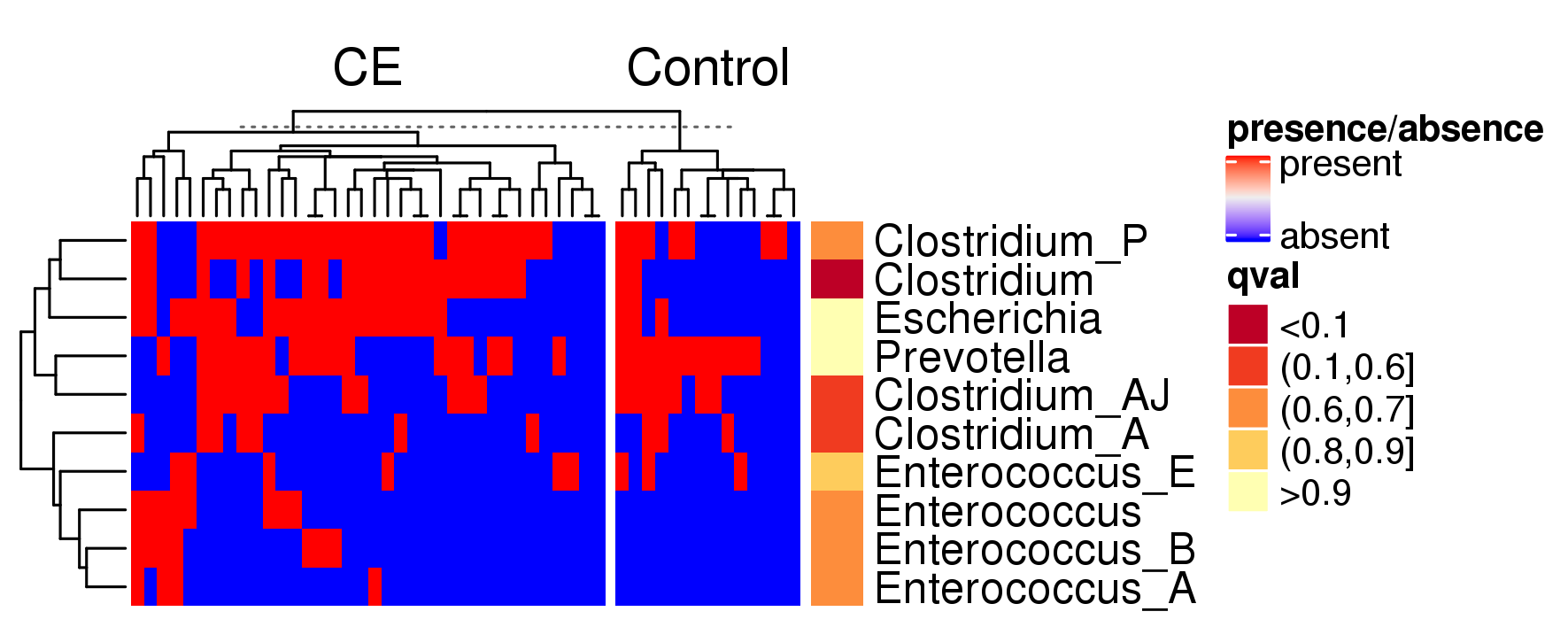

Supplement: Supplementary file 1 — Supplementary Information 1. [file 41598_2022_6576_MOESM1_ESM.png]

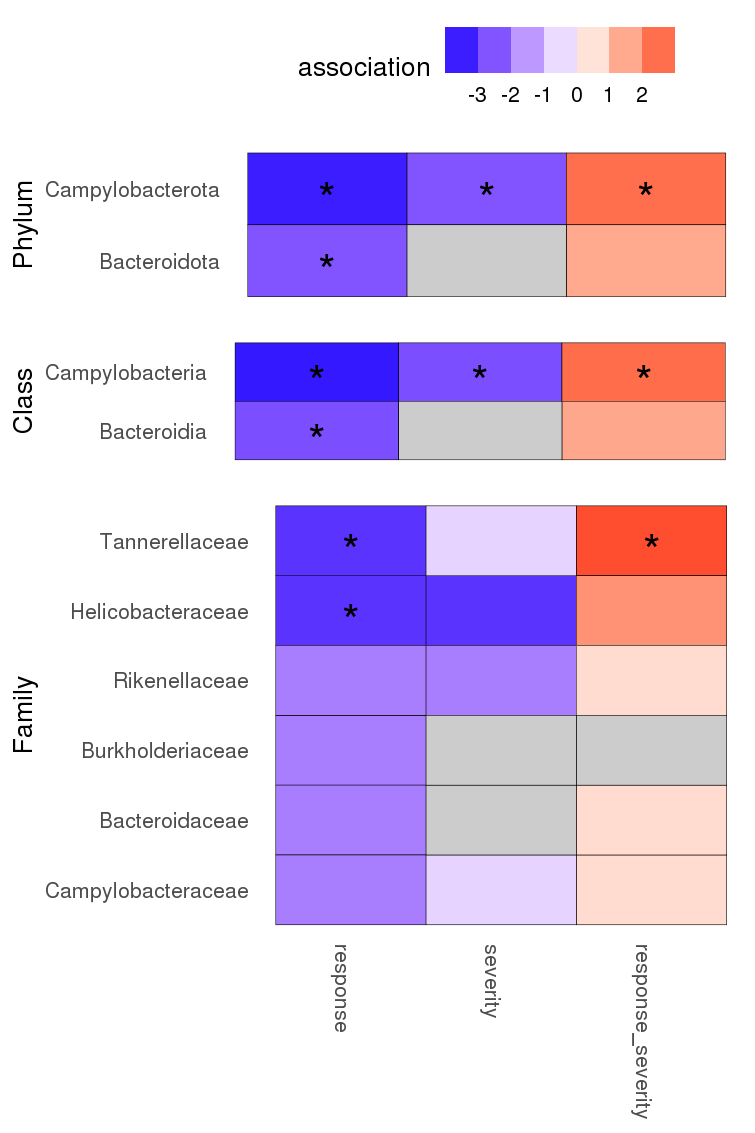

Supplement: Supplementary file 2 — Supplementary Information 2. [file 41598_2022_6576_MOESM2_ESM.png]

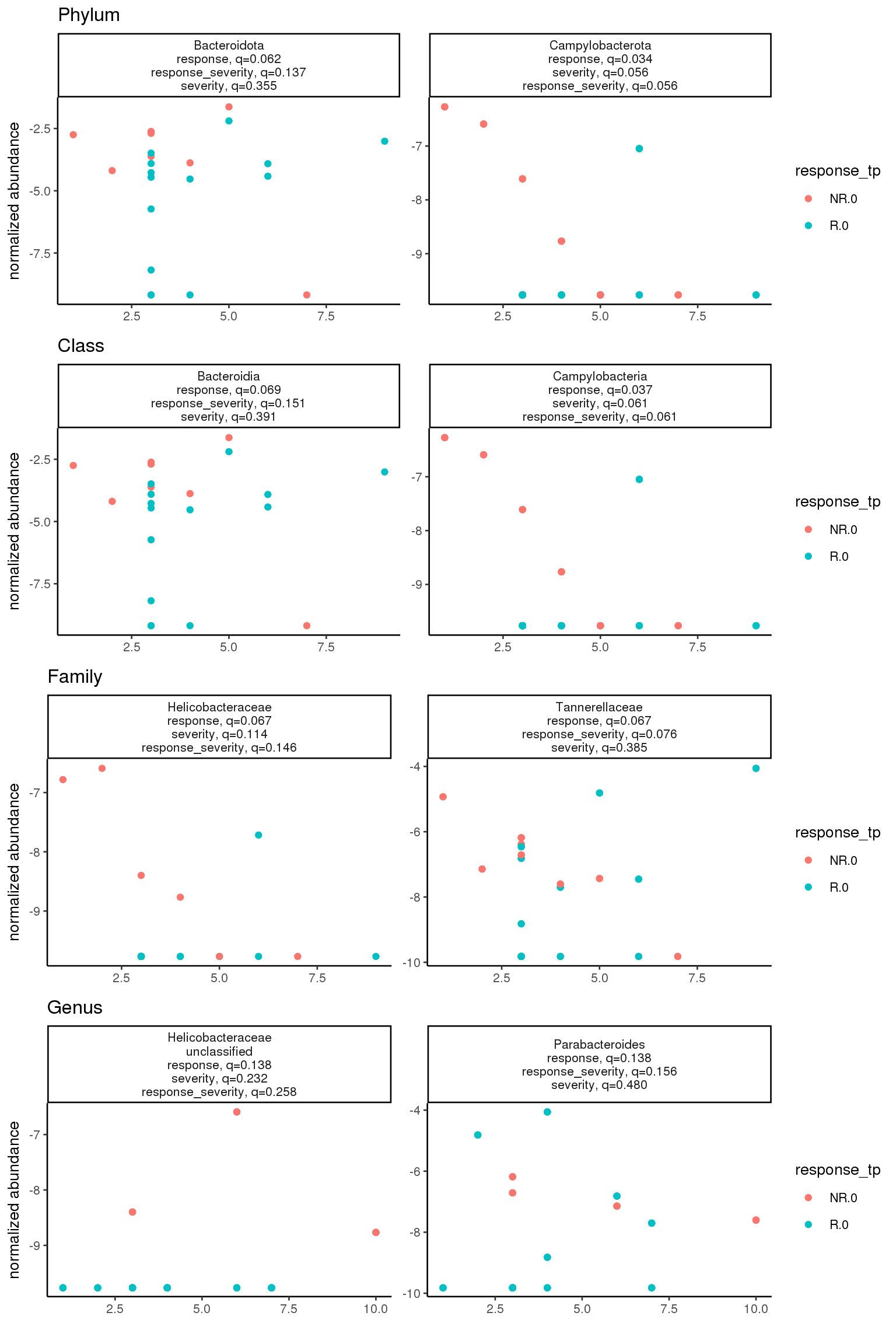

Supplement: Supplementary file 3 — Supplementary Information 3. [file 41598_2022_6576_MOESM3_ESM.png]
