# Supplementary material for: The effect of a hydrolyzed protein diet on the fecal microbiota in cats with chronic enteropathy
Source: Sci Rep. 2022 Feb 17;12:2746. doi: 10.1038/s41598-022-06576-y (PMC8854717; doi:10.1038/s41598-022-06576-y)
Supplement: Supplementary file 4 — Supplementary Information 4. [file 41598_2022_6576_MOESM4_ESM.docx]

Supplementary table 1. Effect of clinical severity (feline chronic enteropathy activity index; FCEAI) on fecal microbiota of cats with suspected or confirmed chronic enteropathy treated with a commercial hydrolyzed protein diet and their clinical response. The linear fixed effect modeling was assessed at every taxonomic level where fixed effects are response, severity (FCEAI), and interaction of two variables (FCEAI_Response). The taxa with significance in either the severity or the response_severity coefficients are: Campylobacterota, Campylobacteria, Tannerellaceae.

| Fixed effect | Tax level | Feature ID | coef | stderr | pval | qval |
| --- | --- | --- | --- | --- | --- | --- |
| response | Phylum | Campylobacterota | 1.7666 | 0.4570 | 0.0011 | 0.0340 |
|  |  | Bacteroidota | 3.3744 | 1.1389 | 0.0083 | 0.0625 |
|  | Class | Campylobacteria | 1.7666 | 0.4570 | 0.0011 | 0.0374 |
|  |  | Bacteroidia | 3.3744 | 1.1389 | 0.0083 | 0.0687 |
|  | Order | Campylobacterales | 1.7666 | 0.4570 | 0.0011 | 0.0850 |
|  |  | Betaproteobacteriales | 2.1941 | 0.7133 | 0.0065 | 0.1221 |
|  |  | Bacteroidales | 3.3744 | 1.1389 | 0.0083 | 0.1250 |
|  | Family | Tannerellaceae | 3.1428 | 0.7445 | 0.0005 | 0.0674 |
|  |  | Helicobacteraceae | 1.5298 | 0.3863 | 0.0009 | 0.0674 |
|  |  | Burkholderiaceae | 2.1941 | 0.7133 | 0.0065 | 0.1456 |
|  |  | Rikenellaceae | 1.9061 | 0.6255 | 0.0069 | 0.1456 |
|  |  | Bacteroidaceae | 2.9373 | 1.1190 | 0.0172 | 0.2806 |
|  |  | Campylobacteraceae | 0.6166 | 0.2423 | 0.0203 | 0.2984 |
|  | Genus | Parabacteroides | 3.1428 | 0.7445 | 0.0005 | 0.1376 |
|  |  | Helicobacteraceae_unclassified | 1.5298 | 0.3863 | 0.0009 | 0.1376 |
|  |  | Alistipes | 1.9061 | 0.6255 | 0.0069 | 0.2579 |
|  |  | Sutterella | 2.1649 | 0.7162 | 0.0073 | 0.2579 |
|  |  | Bacteroides | 4.4095 | 1.4713 | 0.0077 | 0.2579 |
|  |  | Campylobacter | 0.6166 | 0.2423 | 0.0203 | 0.3892 |
|  |  | Enterococcus_A | 1.3424 | 0.5321 | 0.0213 | 0.3892 |
|  |  | DTU089_unclassified | 0.2411 | 0.0962 | 0.0221 | 0.3892 |
|  |  | Enterococcaceae_unclassified | 0.9239 | 0.4005 | 0.0332 | 0.4863 |
| severity | Phylum | Campylobacterota | 1.0539 | 0.3220 | 0.0042 | 0.0559 |
|  | Class | Campylobacteria | 1.0539 | 0.3220 | 0.0042 | 0.0614 |
|  | Order | Campylobacterales | 1.0539 | 0.3220 | 0.0042 | 0.1221 |
|  |  | Enterobacterales | 1.3383 | 0.5878 | 0.0352 | 0.3777 |
|  | Family | Helicobacteraceae | 0.9291 | 0.2721 | 0.0031 | 0.1137 |
|  |  | Rikenellaceae | 1.2695 | 0.4407 | 0.0100 | 0.1828 |
|  |  | Tannerellaceae | 1.2464 | 0.5245 | 0.0288 | 0.3847 |
|  |  | Campylobacteraceae | 0.3810 | 0.1707 | 0.0386 | 0.4050 |
|  | Genus | Helicobacteraceae_unclassified | 0.9291 | 0.2721 | 0.0031 | 0.2320 |
|  |  | Alistipes | 1.2695 | 0.4407 | 0.0100 | 0.2985 |
|  |  | DTU089_unclassified | 0.1872 | 0.0678 | 0.0128 | 0.3212 |
|  |  | Enterococcus | 1.0423 | 0.3883 | 0.0151 | 0.3244 |
|  |  | Parabacteroides | 1.2464 | 0.5245 | 0.0288 | 0.4797 |
|  |  | Campylobacter | 0.3810 | 0.1707 | 0.0386 | 0.4957 |
|  |  | Pseudoflavonifractor | 0.2972 | 0.1352 | 0.0413 | 0.4957 |
| response_severity | Phylum | Campylobacterota | 1.7541 | 0.5576 | 0.0056 | 0.0559 |
|  |  | Bacteroidota | 3.4571 | 1.3895 | 0.0229 | 0.1372 |
|  | Class | Campylobacteria | 1.7541 | 0.5576 | 0.0056 | 0.0614 |
|  |  | Bacteroidia | 3.4571 | 1.3895 | 0.0229 | 0.1509 |
|  | Order | Campylobacterales | 1.7541 | 0.5576 | 0.0056 | 0.1221 |
|  |  | Bacteroidales | 3.4571 | 1.3895 | 0.0229 | 0.2859 |
|  | Family | Tannerellaceae | 3.3813 | 0.9082 | 0.0016 | 0.0763 |
|  |  | Helicobacteraceae | 1.5080 | 0.4712 | 0.0050 | 0.1456 |
|  |  | Rikenellaceae | 1.7514 | 0.7631 | 0.0340 | 0.3850 |
|  |  | Campylobacteraceae | 0.6780 | 0.2956 | 0.0340 | 0.3850 |
|  |  | Bacteroidaceae | 2.9698 | 1.3652 | 0.0432 | 0.4231 |
|  | Genus | Parabacteroides | 3.3813 | 0.9082 | 0.0016 | 0.1557 |
|  |  | Helicobacteraceae_unclassified | 1.5080 | 0.4712 | 0.0050 | 0.2579 |
|  |  | Enterococcus_A | 1.9871 | 0.6491 | 0.0067 | 0.2579 |
|  |  | Enterococcaceae_unclassified | 1.3802 | 0.4886 | 0.0112 | 0.3061 |
|  |  | Bacteroides | 4.8192 | 1.7950 | 0.0151 | 0.3244 |
|  |  | Alistipes | 1.7514 | 0.7631 | 0.0340 | 0.4863 |
|  |  | Campylobacter | 0.6780 | 0.2956 | 0.0340 | 0.4863 |
|  |  | Peptostreptococcaceae_unclassified | 3.3310 | 1.4738 | 0.0364 | 0.4957 |
|  |  | DTU089_unclassified | 0.2583 | 0.1174 | 0.0411 | 0.4957 |

Supplementary table 2. The taxa with significance in either the clinical severity or the response_severity coefficients in cats with suspected or confirmed chronic enteropathy treated with a commercial therapeutic hydrolyzed diet were Campylobacterota, Campylobacteria, Tannerellaceae (sup. Table 1). The table below shows what organisms in our dataset the phyla Bacteroidota and Campylobacterota encompass. Campylobacterota can be traced to two genera: Campylobacter and Helicobactereaceae_unclassified. Bacteroidota is a broader phylum, and encompasses by several organisms at lower taxonomic levels. Notably, Tannerellaceae was a family in the Bacteroidota phylum that was also significant in responder-severity interaction term.

| Phylum | Class | Order | Family | Genus |
| --- | --- | --- | --- | --- |
| Bacteroidota | Bacteroidia | Bacteroidales | Bacteroidaceae | Bacteroidaceae_unclassified |
|  |  |  |  | Bacteroides |
|  |  |  |  | Bacteroides_A |
|  |  |  |  | Bacteroides_B |
|  |  |  |  | F0040 |
|  |  |  |  | Prevotella |
|  |  |  | Bacteroidales_unclassified | Bacteroidales_unclassified |
|  |  |  | Marinifilaceae | Odoribacter |
|  |  |  | Rikenellaceae | Alistipes |
|  |  |  | Tannerellaceae | Parabacteroides |
| Campylobacterota | Campylobacteria | Campylobacterales | Campylobacteraceae | Campylobacter |
|  |  |  | Helicobacteraceae | Helicobacteraceae_unclassified |

Supplementary figure 1. Binary heat map of the presence and absence of fecal bacterial genera in cats with chronic enteropathy (CE) and control cats with no gastrointestinal signs shows prevalence of clostridia, *Escherichia*, and enterococci tend to co-occur in CE cats. Only clostridia, *Escherichia*, enterococci, and *Prevotella* are shown due to their relevance to CE in cats and dogs in the existing literature.

Supplementary figure 2. Effect of clinical severity (feline chronic enteropathy activity index; FCEAI) on fecal microbiota of cats with suspected or confirmed chronic enteropathy treated with a commercial hydrolyzed protein diet and their clinical response. Significance of feature coefficients in linear model were determined at q < 0.05 shown with a star. P-values > 0.05 are set to NA and are colored in grey. Association is -log(q-value)*sign(coefficient). Based on the heat map and the scatter plots, non-responders tend to have a negative relationship with clinical severity (decreasing abundance with increasing FCEAI). On the other hand, there is little or no correlation in abundance and clinical severity in responders.

Supplementary figure 3. The normalized relative abundance at the phylum, class, family and genus level of the significant taxa with respect to clinical severity score (feline chronic enteropathy activity index; FCEAI) in cats with suspected or confirmed chronic enteropathy treated with a commercial hydrolyzed protein diet. Taxa with significant interaction terms show that non-responders have decreasing abundance with increasing severity. On the other hand, abundance in responders is agnostic to CE severity, either because it is absent or because levels remain consistent.
